# Fourteen days free-living evaluation of an open-source algorithm for counting steps in healthy adults with a large variation in physical activity level

**DOI:** 10.1186/s42490-023-00071-9

**Published:** 2023-04-14

**Authors:** Ivar Holm, Jonatan Fridolfsson, Mats Börjesson, Daniel Arvidsson

**Affiliations:** 1 Center for Health and Performance, Department of Food and Nutrition, and Sport Science, Faculty of Education, University of Gothenburg, Gothenburg, Sweden; 2 Center for Health and Performance, Department of Molecular and Clinical Medicine, Institute of Medicine, Sahlgrenska Academy, University of Gothenburg, Gothenburg, Sweden; 3 Sahlgrenska University Hospital, Region Västra Götaland, Gothenburg, Sweden

**Keywords:** Step count, Accelerometer, Algorithms, Open source, Free-living

## Abstract

**Background:**

The number of steps by an individual, has traditionally been assessed with a pedometer, but increasingly with an accelerometer. The ActiLife software (AL) is the most common way to process accelerometer data to steps, but it is not open source which could aid understanding of measurement errors. The aim of this study was to compare assessment of steps from the open-source algorithm part of the GGIR package and two closed algorithms, AL normal (n) and low frequency extension (lfe) algorithms to Yamax pedometer, as reference. Free-living in healthy adults with a wide range of activity level was studied.

**Results:**

A total 46 participants divided by activity level into a low-medium active group and a high active group, wore both an accelerometer and a pedometer for 14 days. In total 614 complete days were analyzed. A significant correlation between Yamax and all three algorithms was shown but all comparisons were significantly different with paired t-tests except for ALn vs Yamax. The mean bias shows that ALn slightly overestimated steps in the low-medium active group and slightly underestimated steps in high active group. The mean percentage error (MAPE) was 17% and 9% respectively. The ALlfe overestimated steps by approximately 6700/day in both groups and the MAPE was 88% in the low-medium active group and 43% in the high active group. The open-source algorithm underestimated steps with a systematic error related to activity level. The MAPE was 28% in the low-medium active group and 48% in the high active group.

**Conclusion:**

The open-source algorithm captures steps fairly well in low-medium active individuals when comparing with Yamax pedometer, but did not show satisfactory results in more active individuals, indicating that it must be modified before implemented in population research. The AL algorithm without the low frequency extension measures similar number of steps as Yamax in free-living and is a useful alternative before a valid open-source algorithm is available.

## Background

Physical activity brings major health benefits by lowering the risk for cardiovascular disease as well as other lifestyle related diseases, depression and musculoskeletal injuries [1]. Both for surveillance and research, it is of importance to measure physical activity, for example to determine compliance to national recommendations and to study the importance of physical activity to health. Number of steps provide important health benefits from physical activity since most of the body is engaged in the activity. The activities performed producing steps, like walking, running or in some sports, are easily accessible for most people [2]. A common way to measure steps in research is with pedometers because they are cheap, small, easy to use [3]. Because of this, pedometers are often used in epidemiological research [3]. Modern pedometers consist of an accelerometer where the information to determine steps is used only. However, accelerometer data can provide additional information such as intensity level, energy expenditure for different activities and even data on sleep [4]. Nevertheless, the number of is steps is still a useful metric because of its simplicity but the accelerometer signal needs to be processed.

A number of factors have to be taken into consideration, including body position and sampling rate, that determines how strong the accelerometer signal is [5]. Further, a frequency filter is applied to reduce noise [6]. Placement at the hip has shown the be the most reliable position to register steps [7]. The most common accelerometer is the ActiGraph with the model GT3X [7], which has its own software called ActiLife (AL) for processing data [8]. However, it is rather expensive and the algorithm for processing data is not accessible. This makes it hard to analyze and understand measurement errors, which is a problem in research. A low frequency extension (lfe) filter has been added to AL to detect activities at a lower intensity. However, ALlfe has shown to have difficulties filtering out noise compared with the normal AL filter (ALn) in free-living, contributing to overestimation of steps compared to a criterion pedometer [9]. Early studies comparing accelerometer and pedometer steps found strong correlation but low agreement, were the accelerometer often reported more steps when using AL than the pedometer [10]. A more recent study in free-living shows a slight underestimation of steps from accelerometer data when using ALn compared with pedometers [11]. Other brands of accelerometers such as the Axivity only provides the raw acceleration data which means that an algorithm for steps needs to be developed.

To determine the true number of steps, visual step counting (direct observation) is considered the golden standard [12]. However, this is only possible in a laboratory setting and not in free-living conditions, in which case a camera attached to the body can be used instead. This is only possible for a few hours due to the discomfort. Therefore, a true free-living golden standard for steps is not yet accessible. Instead, free-living studies are mostly designed for concurrent validity, where two or more devices are compared to determine their agreement.

To get a standardize method for discerning the number of steps from accelerometer data, it necessitates that the algorithm is open source, making it is easy to analyze and share between researchers. Some popular algorithms used for steps in accelerometers are peak detection, autocorrelation, and continuous wavelet transformation (CWT) methods [13]. CWT has shown good accuracy against steps counted by a body camera in semi free-living setting in healthy subjects [14, 15]. Even though these algorithms seem to perform well, they are hard to replicate without a vast programing knowledge since they are not open source. An open-source algorithm based on autocorrelation is available that has been used as part of the rehabilitation for cardiovascular patients and showed low errors compared with manual step count in a controlled setting [12]. Another popular method is peak detection which seems to perform the best overall, when comparing different algorithms across different positions and activities [13]. It scans the signal for peaks over a set threshold and uses different constraints to eliminate false peaks [12]. An open-source algorithm based on peak detection has been presented in a semi free-living setting, with a 95% accuracy when compared to manually counted steps [16] and 89% accuracy in a controlled setting [17]. This algorithm is open source and performs well in a controlled setting, but might have to be further developed before tested in free-living.

A proposed method that is also based on peak detection is an open-source algorithm [18] that is part of GGIR that is a widely used package on processing accelerometer data [19]. It is based on an algorithm that implements constraints to detect and eliminate false steps [20]:

• Periodicity: Time difference between two peaks for the same activity (walking, running etc) is rather fixed since they take place at the same speed. So, a more varying time difference between peaks will be identified as activities other than steps and therefor discarded.
• Similarity: The acceleration for steps should look similar in nature, which means that the peaks in a window look similar or are discarded.
• Continuity: The number of neighboring windows of acceleration surpassing a threshold to form bouts of gait over a certain period.

Compared with a conventional peak detection method, the constraints improved the accuracy by 6.6% for normal walking,9.5% for free walking and 58.9% for data that includes false steps [20] in healthy individuals. The algorithm has also been tested on patients with cardiovascular disease in a controlled setting wearing a accelerometer on the wrist [12], and in 30 participants walking 350 m regular, semi-regular and unstructured with a video recording as reference [21]. Both the studies showed satisfying results. The accuracy of this open-source algorithm has shown promising results, but it still needs to be tested on a larger data set with a variety of activity levels, in a free-living setting over multiple days. It also needs to be compared to other commonly used accelerometer step count methods such as AL before it can be implemented in larger epidemiological studies.

Therefore, the aim of this study was to compare assessment of steps from the open-source algorithm part of the GGIR package and the ALn and ALlfe algorithms to Yamax pedometer (as reference), during free-living over multiple days, in healthy adults with a wide range of physical activity levels.

## Results

Steps from a total of 614 days were used in this study. The participants were categorized into low-medium and high active groups for analysis purpose and are presented in Table 1.

**Table 1:** Participant characteristics

	**Overall**	**Low-medium**	**High**
**Individuals**, n (% female)	46 (54%)	22 (64%)	24 (46%)
**Age**, years, mean (sd)	29 (5)	28 (5)	30 (5)
**Weight**, kg, mean (sd)	72 (15)	76 (20)	68 (8)
**Height**, cm, mean (sd)	175 (10)	172 (11)	178 (9)
**Days of complete data**	614	298	316

Both AL algorithms and Yamax showed strong correlation in both groups, with a slightly stronger correlation between Yamax and ALn (Table 2). The correlation between Yamax and GGIR were strong in the low-medium active group, but lower in the high active group. The Yamax and ALn had similar mean values for steps in both groups with a small overestimation in the low-medium active group and small underestimation in the high active group. In contrast, ALlfe overestimated steps and GGIR underestimated steps in comparison to Yamax in both groups. These differences are also reflected in the large mean absolute percent error (MAPE) for ALlfe versus Yamax in both groups, especially in the low-medium active group. The MAPE for GGIR versus Yamax was instead larger in the high active group.

**Table 2:** Daily steps determined from the different accelerometer algorithms and from the Yamax pedometer in the low-medium and high active groups

Group	Steps	Correlation		Difference		
Algorithm	Mean (sd)	*r*	*p*-value	Mean (sd)	*p*-value	MAPE (%)
**Low-med**						
Yamax	7783 (4248)	-	-	-	-	-
ALn	8037 (4064)	0.88	< 0.001	254 (2078)	0.04	17
ALlfe	14,452 (5474)	0.83	< 0.001	6669 (3050)	< 0.001	88
GGIR	6654 (3978)	0.77	< 0.001	-1129 (2800)	< 0.001	28
**High**						
Yamax	16,773 (7409)	-	-	-	-	-
ALn	16,375 (7324)	0.94	< 0.001	-398 (2645)	0.008	9
ALlfe	23,624 (7962)	0.87	< 0.001	6851 (3886)	< 0.001	43
GGIR	9361 (4199)	0.45	< 0.001	-7412 (6665)	< 0.001	48

*ALn* ActiLife normal filter, *ALlfe* ActiLife low frequency extension, *GGIR* open-source algorithm, *MAPE* mean absolute percent error

**Fig. 1:**
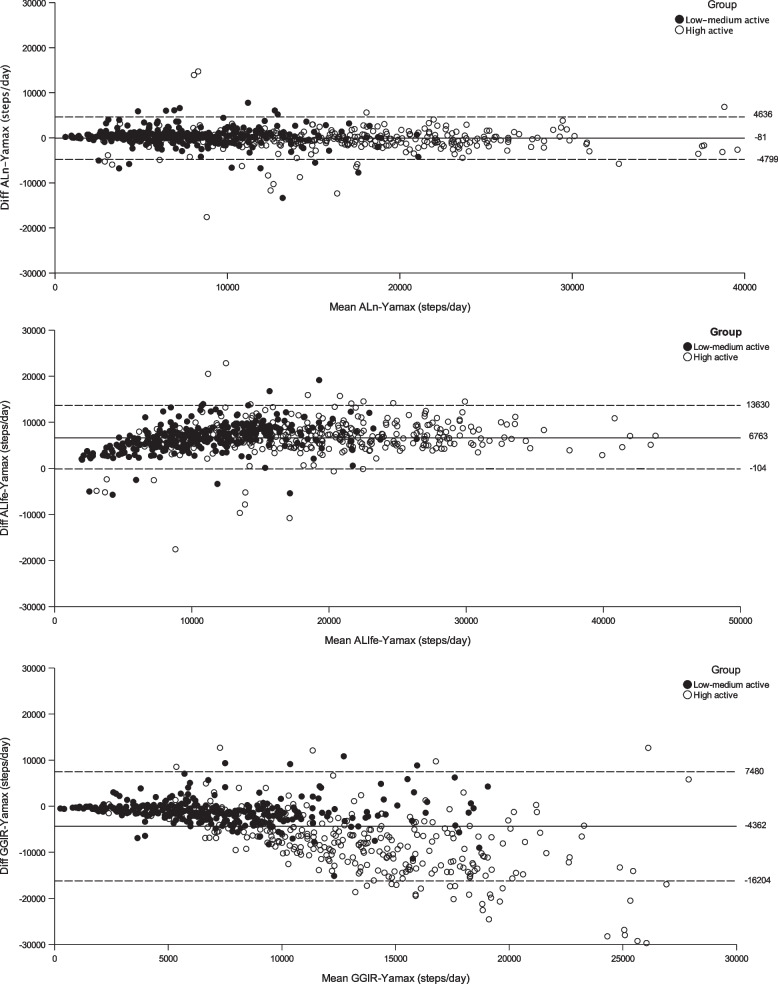
Difference in daily steps for each of the algorithms compared to Yamax across the range of steps. Each circle representing one day. Horizontal lines indicate mean (± 2sd) difference

Altogether, the results confirm a systematic error where ALlfe overestimates and GGIR underestimates the number of steps compared to Yamax. In the case of GGIR, the systematic error was depended on the activity level. This is further highlighted in the Bland–Altman plots presented in Fig. 1, which reveals the distribution of the measurement errors at an individual level. The systematic error and the larger individual variation in the measurement error correspond to the larger MAPE for ALlfe and GGIR compared to ALn. Although the mean difference between ALlfe and Yamax was the same in both groups, the MAPE was larger in the low-medium active group as the error in relation to the total number of steps is larger. The Bland–Altman plot for GGIR versus Yamax visualizes the differential systematic error with increasing underestimation by increasing number of steps. This figure also reveals the larger individual variation in the error in the high active group corresponding to the difference in MAPE between the groups, which confirms a larger random error.

## Discussion

The results of this study shows that the ALn algorithm performs closest to Yamax across the range of physical activity level, with a small measurement error both at group and individual level. This seems to be in accordance with what previous studies have shown in a free-living setting when comparing with a pedometer in a healthy population in the same age span but without regulation in activity level [11]. Hence, ALn is suitable to be used in a variety of populations. The ALlfe largely overestimated by approximately 6700 steps/day compared to Yamax and this systematic error was similar in both groups. That ALlfe tends to generate more steps compared with the other measurements follows the trend in previous studies [9]. ALlfe is made to capture steps in a low active group such as elderly with walking impairments, who are at a much lower activity level than the low-medium group in this study. This means that the results shown here might have been expected since ALlfe records noise at a low frequency which might not be actual steps, noise that the ALn filters out. In addition, there was a large individual variation in the error (random error), especially in the low-moderate active group were the MAPE is 88% compared to 43% in the high active group. When looking at the Bland–Altman plot in Fig. 1 this difference in MAPE can be hard to understand. This is explained in relation to the total number of steps, were individuals in the high active group takes more steps which means that the percentage shown by MAPE becomes smaller. If the individual error would instead be presented as mean absolute error (MAE), the groups would probably be more similar.

The open-source GGIR algorithm underestimates steps compared with Yamax. It is clear that the systematic error is related to activity level where the underestimation becomes larger the higher the step output is. This is highlighted by the mean difference of -1129 in the low-medium group and -7412 in the high active group and the visualization in the Bland–Altman plot in Fig. 1. This is most likely a result by the constraints put in the algorithm to remove false steps. When the step frequency increases, too many real steps are filtered out. Since most participants in the high active group are long distance runners it is possible that the GGIR algorithm have problems detecting activities such as running which could explain the discrepancy between the algorithms in the high active group. This makes sense since the GGIR algorithm has only been developed for walking at different speeds and not for running [20]. The individual error (random error) is high across both groups with a MAPE of 48% in the high active and 28% in the low-medium. However, since the low-medium group still can be physically active up to 3 h per week, it is possible that the measurement error is related to activities of higher intensities such as running in this group as well.

A previous study of cardiac rehabilitation showed a MAPE of 4% in normal walking and 11% in running when comparing the GGIR algorithm to visual step count [12], which is much lower than our study shows. However, the participants are not the same where one could imagine that the cardiac patients take smaller and slower steps compared with healthy adults. The periodicity constraint regarding time intervals between peaks to identify bouts of steps is set to a time threshold to eliminate false steps. This time threshold is set in relation to steps while walking [20]. Therefore, we suggest that the threshold is changed to include peaks closer together to capture higher intensity activities such as running. If the time threshold is expanded, it might lead to more false steps recorded, but the similarity and continuity constraints are in place as well to help address and remove false steps withing the threshold of the periodicity constrain which means that this should not be a problem.

## Strengths and limitations

To our knowledge, no other study has evaluated open-source algorithms for counting steps in free-living over such an extended number of days. Another strength in this study is the number of participants covering a large span of activity levels. Because the Yamax pedometer is rather large, the participants were instructed to take it off during the night, which they did not do with the accelerometers. This means that if the participants went up during the night to for example drink water or go to the bathroom, those steps were not recorded by the pedometer. When running the algorithms on the accelerometer data, it is hard to remove a specific number of hours to compensate for this difference. Consequently, steps taken during the night, for example going to the bathroom or kitchen, were not registered by the pedometer. There is no golden standard method for counting steps in free-living over an extended amount of time, but the Yamax pedometer is often used in research and was therefore chosen as the reference method in this study. Because of this, we can only say how the algorithms in question perform in relation to Yamax as concurrent validity.

## Conclusion

This study shows that the GGIR open-source algorithm captures steps fairly well in a low-medium active group when compared with the Yamax pedometer but does not show satisfactory results on higher activity levels, which means that the algorithm must be modified before implemented in a larger variation of populations. The ALn algorithm measures steps close to the Yamax pedometer in free-living and is therefore a suitable alternative for measuring steps in free-living before a valid open-source algorithm is available.

## Methods

### Study design

The study is part of the methodological project Measuring Energy expenditure and Diary intake at different Activity Levels (MEDAL) and evaluated the accuracy of one open-source and two closed-source algorithms to determine steps from accelerometer data collected at the right hip during free-living for two weeks. The Axivity AX3 accelerometer (Axivity Ltd., Newcastle upon Tyne, UK) with a sampling rate of 100 Hz and range of 8 g and the Yamax SW-200 Digi-Walker Pedometer (Yamax, Bridgnorth, UK) were used in this study. The Yamax SW-200 has demonstrated good accuracy when measuring steps in free-living [22] and was used as reference. The number of steps from the pedometer was logged by the participants for each day in an activity diary. The participants could also write down if they for some reason removed the monitors and what type of physical activity they performed during the day and for how long. The pedometer was taken off during the night, but the accelerometer was worn for 24 h.

### Participant characteristics

Participants between 18–40 years old were recruited by distributing leaflets at Gothenburg university, from Facebook online advertisement and from local running sports clubs. Data was collected from 46 participants including 25 females and 21 males. The MEDAL project targeted individuals with a large variation in physical activity and recruited participants into a low-moderate active group (< 150 min/week of vigorous physical activity) and high active group (> 300 min/week of vigorous physical activity). Further, individuals with mainly non-ambulatory activities not including steps, such as cycling, swimming or strength training, were excluded. This means that the high active participants in the study mainly consisted of runners but also some football players. This study has been approved by the Swedish Ethical Review Authority (2019–05,316, 2020–00,010) and has been performed in accordance with the Declaration of Helsinki. The participants provided informed consent to participation. One male and one female in the low-medium active group chose to withdraw from participation after the first week and the first day, respectively.

### Algorithms

An open-source algorithm and two closed-source algorithms was used to calculate steps from the accelerometers. The open-source algorithm is based on the peak detection method, which seems to be the most accurate way to measure steps when compared with other commonly used methods [13]. This peak detection algorithm is a part of the GGIR package [19] and the detailed information on how to run the algorithm can be found [18]. When comparing with other open-source algorithms, there are several reasons why choosing this algorithm:

• GGIR is a well-known package when it comes processing accelerometer data, which means that many researchers use it
• The algorithm is based on a peak detection method and addresses the problem with detecting false steps by adding constraints: Periodicity, similarity, and continuity
• The algorithm is designed to work on different locations on the body and has been tested in laboratory and semi-free-living setting in several studies [12, 20, 21]
• This algorithm provides better step count accuracy and fewer false steps when compared with a normal peak detection method [20]
• It has been tested on patients with cardiovascular disease as well as healthy participants [12, 20]
• It is free, easily accessible, and ready to be used with minimal programing knowledge

Accelerometer data were also processed in the AL software (ActiGraph LCC, Pensacola, FL, USA) with the normal filter (ALn) and the low frequency extension filter (ALlfe) [8].

### Statistical analysis

The data for each participant consisted of four measures of number of steps from each day. One from the Yamax pedometer and one for each of the algorithms used to calculate steps from the accelerometer. Days which had missing data from at least one of the algorithms were removed from analysis. This resulted in 28 days or 4% of the data removed. Two outlier days from the same participant were identified as technical error based on the large difference (> 45,000 steps) and removed from analysis. The statistical evaluation included Pearson correlation, mean (sd) difference accompanied with paired t-tests to determine measurement bias, mean absolute percent error (MAPE) for the absolute size of error, and Bland–Altman plots for visualization of different components of measurement errors, i.e. systematic error (differential or non-differential) and random error (individual variation).

## Data Availability

The datasets used and/or analyzed during the current study are available from the corresponding author on reasonable request.
